# Collateral flow and pulsatility during large vessel occlusions: insights from a quantitative *in vitro* study

**DOI:** 10.3389/fbioe.2024.1421714

**Published:** 2024-07-17

**Authors:** Claudio A. Luisi, Omid Nikoubashman, Ulrich Steinseifer, Martin Wiesmann, Michael Neidlin

**Affiliations:** ^1^ Department of Cardiovascular Engineering, Institute of Applied Medical Engineering, Medical Faculty, RWTH Aachen University, Aachen, Germany; ^2^ Clinic for Diagnostic and Interventional Neuroradiology, Medical Faculty, RWTH Aachen University, Aachen, Germany

**Keywords:** anatomical phantom model, circle of Willis, collateral flow, hemodynamics, ischemic stroke, large vessel occlusion, neurovascular intervention, particle image velocimetry

## Abstract

Acute ischemic stroke caused by large vessel occlusions is being increasingly treated with neurovascular interventions. The hemodynamics within the collateral system of the circle of Willis (CoW) hemodynamics play a fundamental role in therapy success. However, transient *in vivo* data on pathological collateral flow during large vessel occlusions are not available. Moreover, there are no flow models that accurately simulate the hemodynamic conditions in the CoW during large vessel occlusions. We used a circulatory loop to generate highly reproducible cerebrovascular-like flows and pressures and used non-invasive flow visualization and high-resolution flow and pressure measurements to acquire detailed, time-dependent hemodynamics inside an anatomical phantom of the CoW. After calibrating a physiological reference case, we induced occlusions in the 1. middle cerebral artery, 2. terminal carotid artery, and 3. basilar artery; and measured the left posterior communicating artery flow. Mean arterial pressure and pulse pressure remained unchanged in the different occlusion cases compared to the physiological reference case, while total cerebral flow decreased by up to 19%. In all three occlusion cases, reversed flow was found in the left posterior communicating artery compared to the reference case with different flow magnitudes and pulsatility index values. The experimental results were compared with clinical findings, demonstrating the capability of this realistic cerebrovascular flow setup. This novel cerebrovascular flow setup opens the possibility for investigating different topics of neurovascular interventions under various clinical conditions in controlled preclinical laboratory studies.

## 1 Introduction

Acute ischemic stroke is one of the leading causes of death and disability worldwide (Donkor, 2018). The treatment options of acute ischemic stroke due to large vessel occlusions have shifted toward endovascular thrombectomy as the emerging standard. To date, stent-retriever thrombectomy and aspiration thrombectomy with distal access catheters are the most common techniques, while balloon guide catheters are used more frequently (Chueh et al., 2020). A common feature of all the different mechanical recanalization procedures is that suction is applied to at least one of the two aspiration and access catheters in order to reverse the local blood flow and prevent thrombus fragments from floating off during thrombectomy (Boisseau et al., 2020). Overall, endovascular thrombectomy is performed via different techniques on diverse cerebrovascular anatomies at various flow and pressure conditions. Regardless of the specific technique and patient’s pathophysiology, optimized blood flow control during endovascular thrombectomy is required for the procedure to be effective and safe (Lally et al., 2016; Schönfeld et al., 2020).

The evaluation of the different techniques of endovascular thrombectomy, however, are rather based on clinical experience from single centers (Munoz et al., 2023). The most common *in vivo* animal model allows conclusions to be drawn about thrombectomy procedures (Gralla et al., 2006), but it does not provide insights into collateral blood flow, which has been described as a contributing factor for endovascular thrombectomy success in humans (Lally et al., 2016). Collaterals of the human circle of Willis (CoW), such as the posterior communicating artery (PComA), can reroute blood flow after occlusion in order to maintain the blood flow (Liebeskind, 2014). The physiological PComA flow was investigated through both color-coded duplex sonography measurements in terms of direction and peak velocity (Klotzsch et al., 1996) as well as three-dimensional phase contrast magnetic resonance imaging in terms of velocity field and volumetric flow (van Ooij et al., 2013). In cases of middle cerebral artery (MCA) occlusions, the PComA status is important for a better outcome trend (Sadeh-Gonik et al., 2023), and there are indications of flow rerouting in the ipsilateral PComA in MCA stroke (Liebeskind et al., 2016); however, no *in vivo* data exist on the time-dependent PComA flow during large vessel occlusions and thrombectomy.

As an alternative to *in vivo* studies, *in vitro* and *in silico* models of ischemic stroke and endovascular thrombectomy have been established in the past by several research groups (Luraghi et al., 2021a). On the experimental side, researchers have evaluated stent-retriever thrombectomy (Madjidyar et al., 2015) and aspiration thrombectomy at physiological pulse pressure and flow conditions in truncated cerebrovascular models (Madjidyar et al., 2021). Several *in vitro* thrombectomy studies have been performed using peristaltic pumps, generating either high heart rates (Chueh et al., 2013) or weak-pulsatile flow conditions (Fitzgerald et al., 2021; Nogueira et al., 2022); thus, there is still a need for realistic cerebrovascular flow models. On the computational side, continuum models on CTA-derived cerebrovascular anatomies have been used to study stent-retriever thrombectomy (Luraghi et al., 2021b) and aspiration thrombectomy (Neidlin et al., 2016); furthermore, increasing modeling complexity was added for including thrombus characteristics (Fereidoonnezhad et al., 2021) in order to improve the prediction accuracy and significance of the simulations. Nevertheless, in both the *in vitro* and *in silico* studies, simplifications and assumptions about the hemodynamic conditions during ischemic stroke were made, although collateral flows play a fundamental role in therapy success. However, transient *in vivo* data on pathological collateral flow during large vessel occlusions are not accessible for validation. Moreover, there are currently no experimental flow models available that simulate realistic flow and pressure conditions in the CoW during large vessel occlusions. Realistic cerebrovascular flow models are necessary for studying neurovascular interventions under controlled conditions and optimizing the blood flow control during endovascular thrombectomy in order to improve the treatment for acute ischemic stroke.

To close this gap, we developed an advanced experimental setup, which reproduced the cerebral circulation within an anatomical cerebrovascular phantom and allowed the investigation of the time-dependent collateral flow. Our objective was to demonstrate the feasibility of gaining quantitative information on the hemodynamics in the CoW, including collateral flow and pulsatility, during large vessel occlusions under controlled conditions. To achieve this objective, we introduced a realistic cerebrovascular flow model, generated highly reproducible flows and pressures, and acquired the hemodynamic parameters of the afferent and efferent vessels of a cerebrovascular phantom with a high temporal resolution. The PComA flow profile, its direction, and pulsatility were determined with a high spatial and temporal resolution through particle image velocimetry (PIV).

## 2 Materials and methods

For this study, we synchronized the non-invasive PIV technique to a circulatory loop in order to gain quantitative details of the communicating flow in a cerebrovascular phantom over the cardiac cycle for a physiological reference case and during three common large vessel occlusion cases. The feasibility of both generating physiological conditions in the cerebral circulation and performing PIV measurements within is explained in Luisi et al. (2023); however, several of the stated limitations were overcome in this study through the following factors:

1. Improved robustness of the circulatory loop: stable total artificial heart and tunable aortic compliance;
2. New cerebrovascular phantom: without material defects and averaged vessel diameters from a cohort of 100 anonymized patients;
3. Superior PIV setup: higher spatial resolution (increased by factor 1.8) for visualizing the flow in the smallest communicating artery
4. Enhanced data acquisition: higher frequency (increased by factor 10) of flow and pressure measurement, as well as a synchronized, phase-locked PIV measurement enabling a transient analysis

**FIGURE 1:**
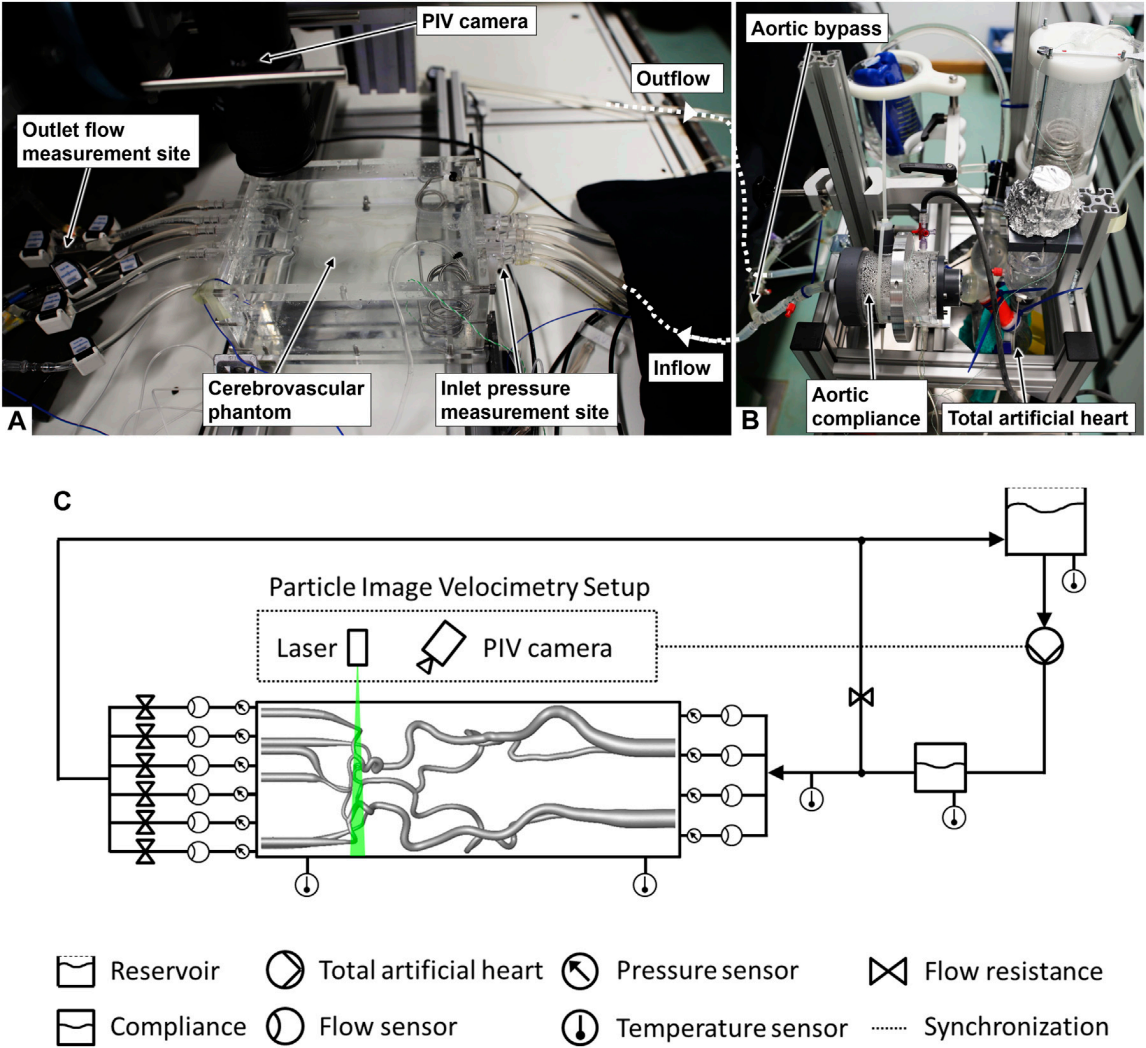
Images of the experimental setup: **(A)** measurement sites and cerebrovascular phantom (contours hardly visible due to the refractive index matching of the phantom material and the fluid), **(B)** circulatory loop, and **(C)** scheme of the setup (adapted from Luisi et al. (2024), used under CC BY 4.0).

The experimental setup with the measurement system and the circulatory loop are presented in Figure 1.

### 2.1 Circulatory loop

We generated the hemodynamic boundary conditions of the anatomical cerebrovascular phantom through a circulatory loop. A total artificial heart (ReinHeart TAH 2.0, ReinHeart TAH GmbH, Aachen, Germany) generated a highly reproducible flow and pressure set to 40%:60% for the ratio of duration of systole to diastole at a frequency of 60 beats per minute as the baseline case. Physiological flow curves at the afferent vessels of the cerebrovascular phantom were achieved by the use of an aortic compliance, and an aortic bypass was adjusted targeting the physiological total cerebral blood flow (tCBF). Downstream flow resistance elements (see Figure 1C) were adapted only for the physiological reference case in order to match the arterial flow fractions of the comparing *in vivo* data from the literature. After adapting to the reference case, the settings of the flow resistance elements remained the same for all large vessel occlusion cases.

A transparent blood analog was used for the test loop made of a water–glycerol mixture kept at 45°C ± 1°C with a dynamic viscosity of 4.01 ± 0.02 mPa measured using a cone-plate rheometer and a density of 1.137 g mL^-1^ at the nominal temperature (Cheng, 2008). The difference in the refractive indices was approximately 0.001 between the fluid and phantom material at the nominal temperature. The anatomical phantom was suspended in the same fluid for modeling the surrounding brain tissue with up to 7 mmHg intracranial pressure on the posterior circulation while having a dampening effect on the oscillations of the phantom during the cardiac cycle.

### 2.2 Cerebrovascular phantom

The cerebrovascular phantom model used in this study is based on the geometry file published in Luisi et al. (2024). In summary, computed tomography angiography (CTA) scans of 100 anonymized patients’ cerebral arteries were used to determine average vessel diameters for the cerebrovascular anatomy. Data handling was approved by the Ethics Review Committee of the University Hospital RWTH Aachen. The number of ethics approval is 335-15.. A cohort of 100 patients was required to avoid patient-specific variabilities in vessel diameters in order to be consistent with population-based *in vivo* results of flows and pressures from the literature, which were used for the cerebrovascular flow model calibration. The afferent vessels of the cerebrovascular phantom were the left and right vertebral arteries at the V1 segment (LVA and RVA) and left and right internal carotid arteries at the C1 segment (LICA and RICA). Efferent vessels of the phantom model reached the A2 segment of the left and right anterior cerebral arteries (LACA and RACA), the M1 segment of the left and right middle cerebral arteries (LMCA and RMCA), and the P2 segment of the left and right posterior cerebral arteries (LPCA and RPCA). Vessels of the cerebrovascular phantom were fully developed and without stenoses. The cerebrovascular phantom had a complete CoW anatomy with bilateral posterior communicating arteries (LPComA and RPComA) and the presence of an anterior communicating artery (AComA). The model diameter of the LPComA was between 1.57 mm and 1.61 mm, while the RPComA diameter was between 1.83 mm and 1.86 mm. Instructions of the manufacturing process for creating this type of flexible and translucent anatomical phantoms were described in Luisi et al. (2023). The elasticity of the phantom material, a two-component silicone rubber (RT 625 A/B, Wacker Chemie AG, Köln, Germany), was approximately 0.53 MPa, which is determined by Young’s modulus at normal temperature and pressure, close to the result from a human MCA *postmortem* study (0.42 MPa) (Chueh et al., 2009). The compliance of the phantom model was 0.0146 ± 0.0001 mL mmHg^-1^ within the tested pressure range at normal temperature.

### 2.3 Data acquisition and processing

Volumetric flows of the afferent and efferent arteries were measured with a set of ultrasound flow sensors (Transonic Systems Inc., Ithaca, USA). Static pressures of the afferent and efferent arteries were measured using pressure transducers (Xtrans, CODAN pvb Medical GmbH, Lensahn, Germany) at the connectors to the phantom (see Figures 1A, C). The data acquisition system ensured simultaneous and a 1-kHz time-resolved flow and pressure data recording, and was synchronized with the optical communicating artery flow measurement. The mean arterial pressure (MAP) was gained from the static pressure measured at the RICA. The pressure measurement had an estimated accuracy of ±0.5 mmHg, while the ultrasound flow measurement had an estimated accuracy of 6% within the linearity range specified by the device.

The communicating artery flow field was measured inside the transparent phantom via PIV, synchronized with the data acquisition system (phase-locked PIV). The PIV system consisted of a double-pulse laser (EverGreen70, Quantel, Les Ulis, France) with a wavelength of 532 nm, seeding particles with 10.5 ± 2 µm diameter, and a camera with a 4-megapixel CCD sensor (FlowSenseEO 4M-32, Dantec Dynamics, Skovlunde, Denmark). A resolution of 0.009 mm per pixel was achieved with the used lens (Micro-Nikkor 60mm f/2.8D, Nikon, Tokyo, Japan). The measurement plane in the center of the communicating artery was acquired for 150 cardiac cycles and with 1,200 images for each case. The volumetric flow of the posterior communicating artery was obtained through mathematical integration of the velocity field over the vessel diameter, assuming rotational symmetry along the vessel centerline, and cubic spline interpolation along time. Volumetric flows and standard deviations for each vessel were determined by superposition of all cardiac cycles, and the corresponding flow pulsatility index (PI) was derived according to Eq. 1:

$$\text{PI}=\frac{{\Delta Q}_{\text{systolic}↔\text{diastolic}}}{\left|Q_{\text{mean}}\right|}.$$ (1)

where the flow amplitude between systolic and diastolic flows is represented by ΔQ_systolic↔diastolic_ and the absolute value of the mean flow is represented by Q_mean_. The PIV-derived flow measurement uncertainty was estimated to be less than 6 mL min^-1^.

### 2.4 Investigated cases

First, a physiological case was created by calibrating flows and PI values of the afferent and efferent vessels of the CoW to available *in vivo* data from the literature (Zarrinkoob et al., 2015; Correia de Verdier and Wikström, 2016; Zarrinkoob et al., 2016). Then, different large vessel occlusion cases were induced through the clamping of the vessels for a hemodynamic study of the collateral flows:

1. Occlusion of the M1 segment of the LMCA.
2. Occlusion of the intersection of LMCA M1, LACA A1, and terminal LICA, known as the left carotid-T occlusion.
3. Basilar artery (BA) occlusion.

**FIGURE 2:**
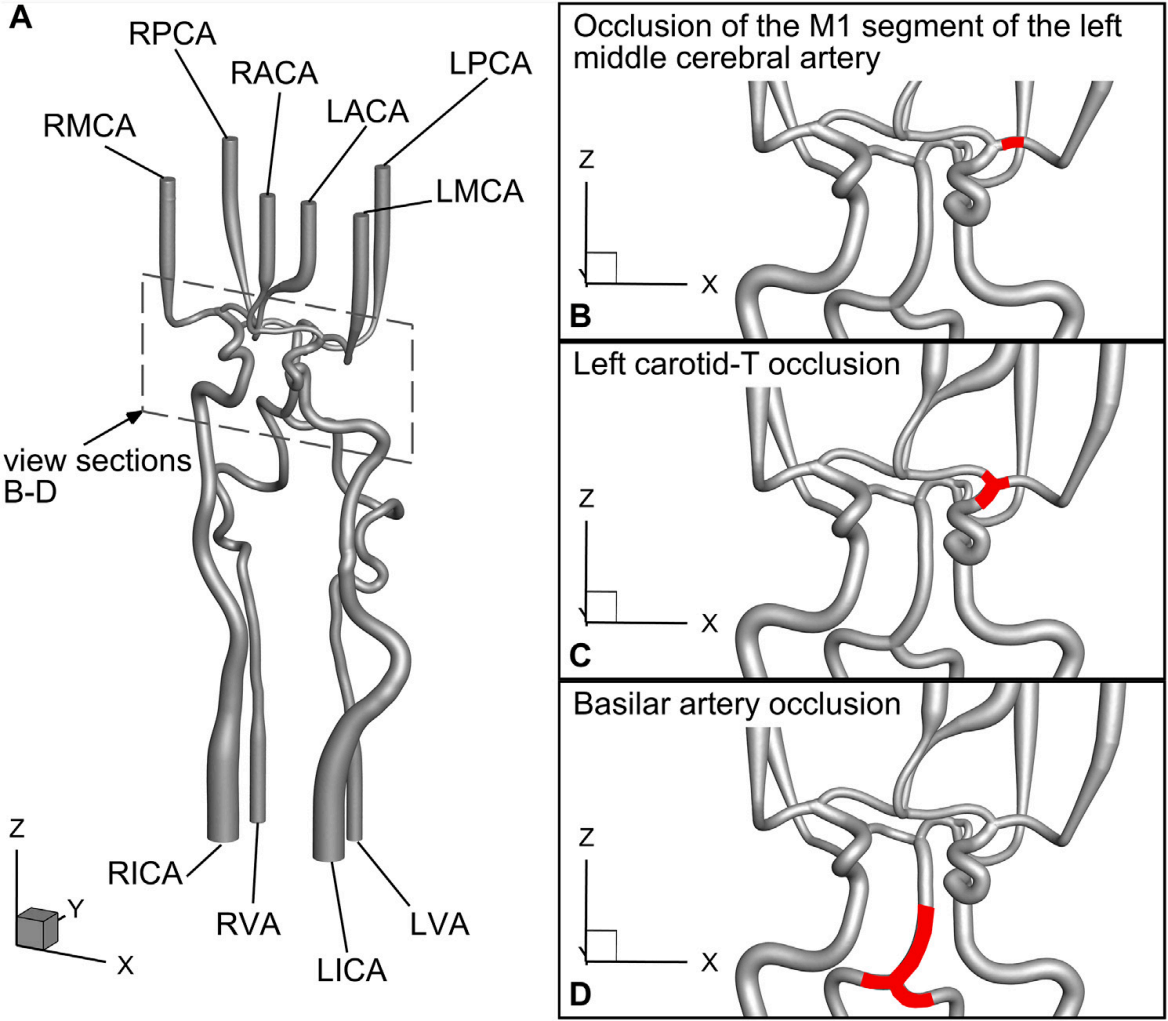
Detailed schematic view of the geometrical model drawn from the geometry file published in Luisi et al. (2024) and the investigated large vessel occlusion cases: **(A)** naming of the vessels, **(B)** case 1: occlusion of the M1 segment of the left middle cerebral artery, **(C)** case 2: left carotid-T occlusion, and **(D)** case 3: basilar artery occlusion.

An overview of vessel naming is shown in in Figure 2A, and a schematic view of the large vessel occlusion cases is presented in Figures 2B–D. During the experiments, time-resolved data on flows and static pressures for all afferent and efferent vessels, as well as the spatially resolved PIV images of the LPComA, were recorded simultaneously.

## 3 Results

**FIGURE 3:**
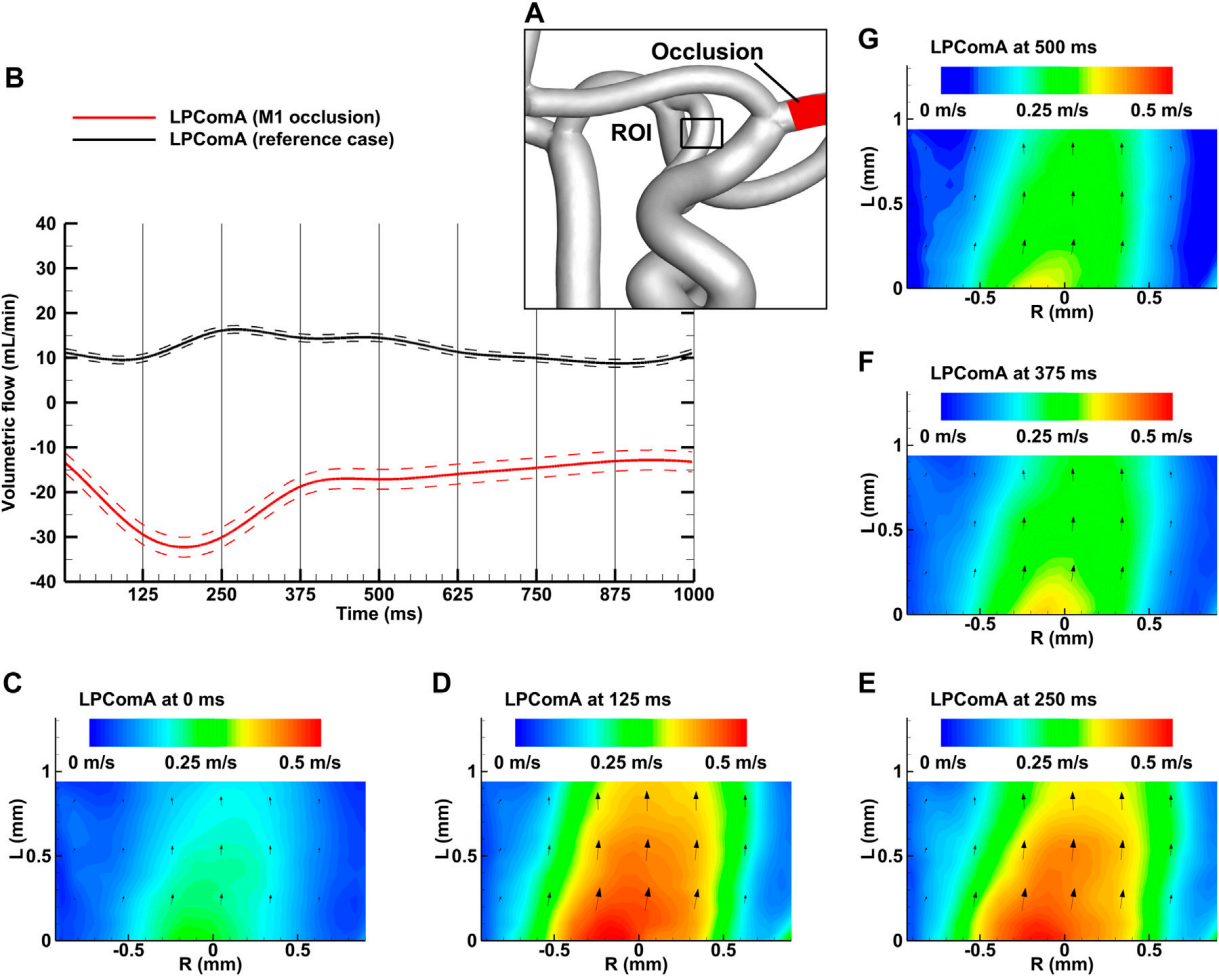
Left M1 occlusion case for the complete CoW anatomy. **(A)** Region of interest (ROI) of the LPComA used for the PIV image acquisition; **(B)** LPComA flow for the M1 occlusion and the reference case over one cardiac cycle at 60 beats per minute averaged from 150 cardiac cycles. Solid lines: transient mean flows; dashed lines: standard deviation range of flows. **(C–G)** Velocity field inside the LPComA in the radial direction R and axial direction L at specific times obtained via phase-locked PIV images and averaged over the total 150 cardiac cycles.

**FIGURE 4:**
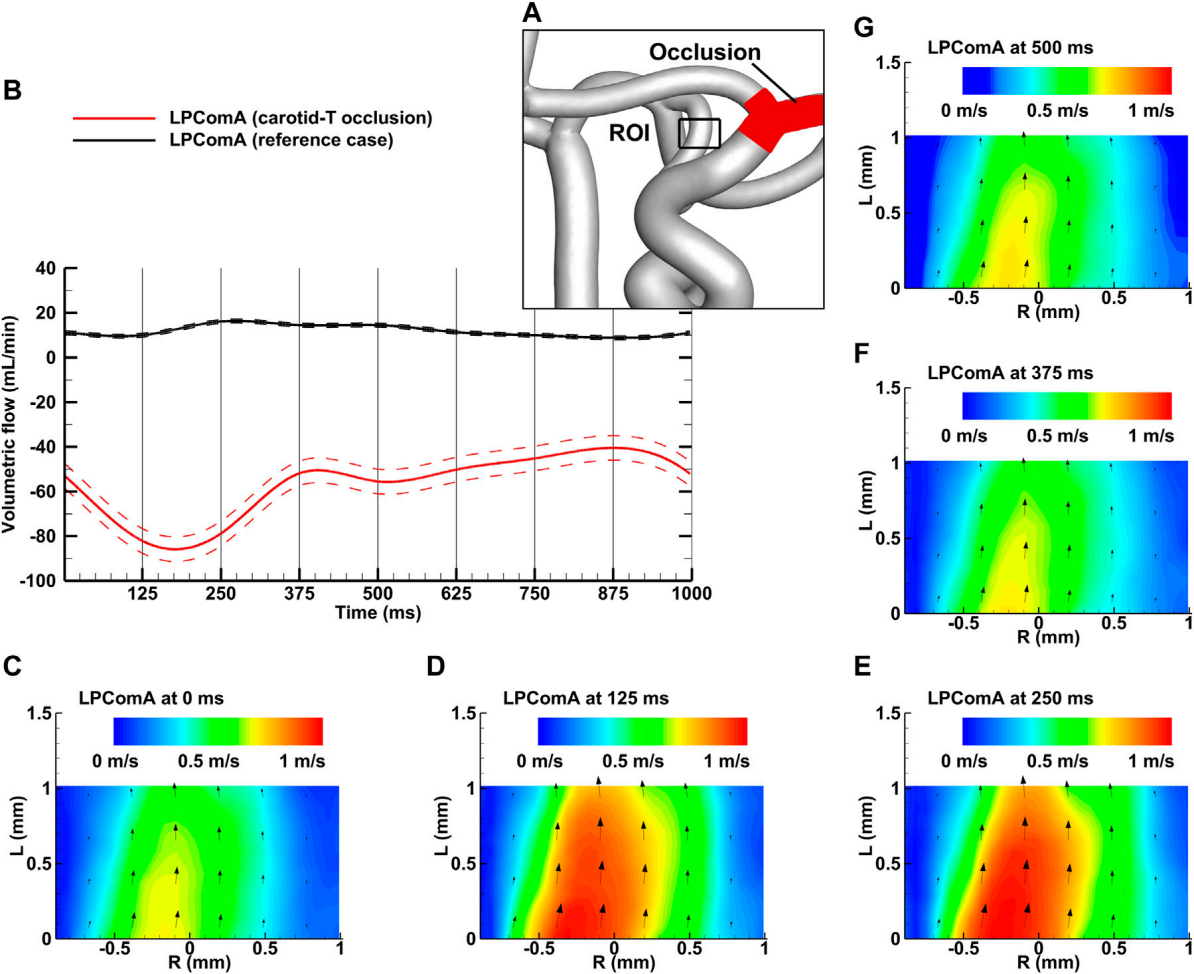
Left carotid-T occlusion case for the complete CoW anatomy. **(A)** Region of interest (ROI) of the LPComA used for the PIV image acquisition; **(B)** LPComA flow for the carotid-T occlusion and the reference case over one cardiac cycle at 60 beats per minute averaged from 150 cardiac cycles. Solid lines: transient mean flows; dashed lines: standard deviation range of flows. **(C–G)** Velocity field inside the LPComA in the radial direction R and axial direction L at specific times obtained via phase-locked PIV images and averaged over the total 150 cardiac cycles.

**FIGURE 5:**
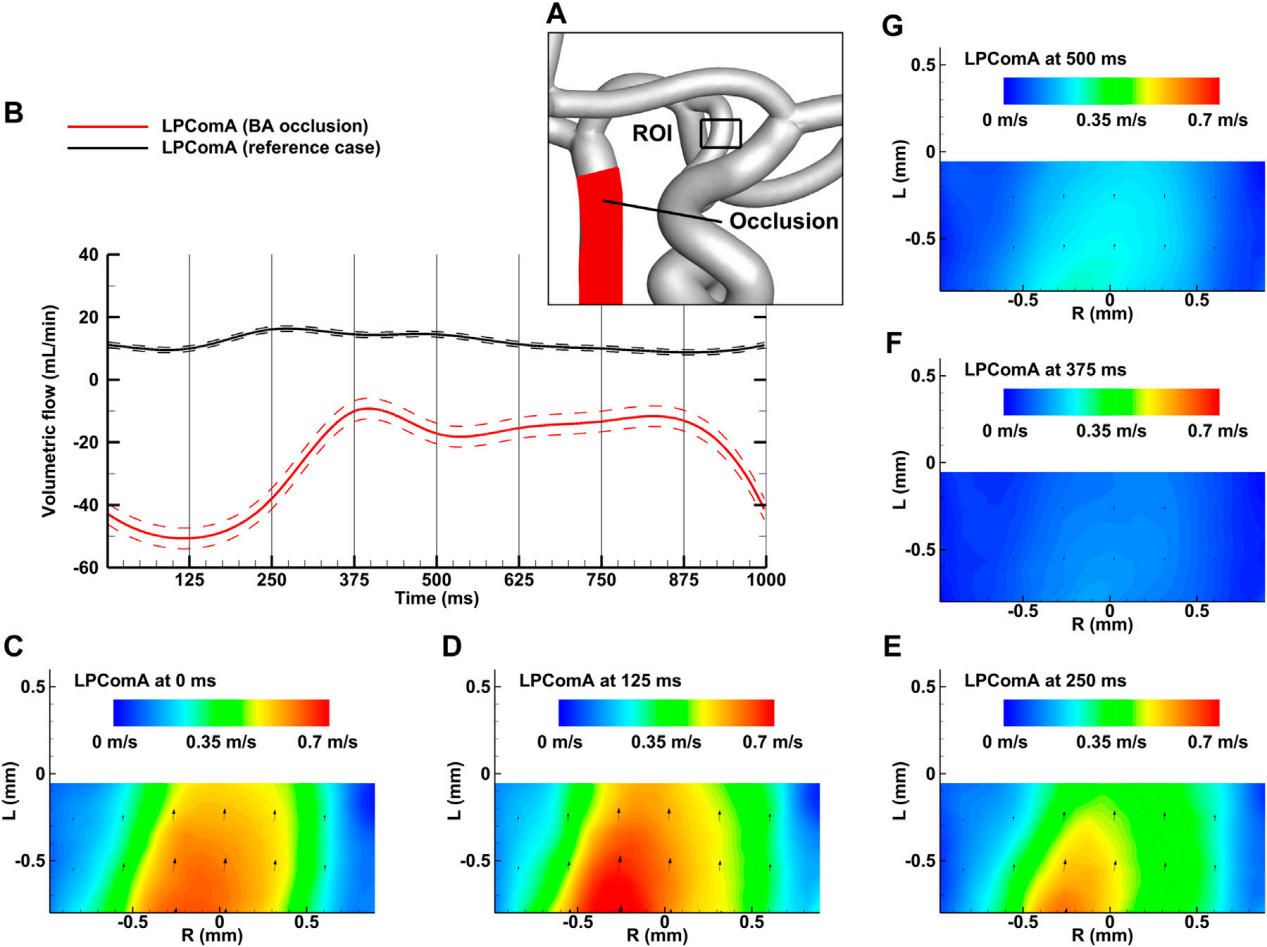
BA occlusion case for the complete CoW anatomy. **(A)** Region of interest (ROI) of the LPComA used for PIV image acquisition; **(B)** LPComA flow for the BA occlusion and the reference case over one cardiac cycle at 60 beats per minute averaged from 150 cardiac cycles. Solid lines: transient mean flows; dashed lines: standard deviation range of flows. **(C–G)** Velocity field inside the LPComA in the radial direction R and axial direction L at specific times obtained via phase-locked PIV images and averaged over the total 150 cardiac cycles.

In the physiological reference case without vessel occlusions, the mean total cerebral blood flow (tCBF) was 647.4 ± 6.7 mL min^-1^ at the MAP of 101 ± 2 mmHg with the pulse pressure of 78 ± 1 mmHg. Flow fractions for the single vessels are listed in Table 1. The LPComA flow was retrograde for the physiological reference case, flowing unidirectional from LPCA to LICA, with 12.0 ± 0.9 mL min^-1^ and a PI value of 0.63 ± 0.10. The measured systolic velocity peak for the physiological reference case was 0.18 m s^-1^, and the systolic velocity averaged over the vessel diameter was 0.13 m s^-1^. In the subsequent Figures 3–5, volumetric flows in the LPComA are depicted for each occlusion case in comparison to the physiological reference case over one cardiac cycle, and spatially resolved velocity fields in the LPComA are presented at specific times.

**TABLE 1:** Vessel mean flows, flow fractions, and flow pulsatility indices (PI) are shown for the physiological reference case obtained in this study on the left side. The comparative *in vivo* data from the literature are shown on the right-hand side with additionally calculated flow fractions, which relate the vascular flow to the corresponding sum of either inflows or outflows and are not included in the cited literature. The reported total cerebral blood inflow is 657 ± 94 mL min^-1^ (Zarrinkoob et al., 2015; Zarrinkoob et al., 2016), and mean flows and PI values are indicated with standard deviation. The total cerebral blood outflow reported in the literature (Correia de Verdier and Wikström, 2016) is 698 mL min^-1^ in total, with mean flows and PI values presented with the value range.

		Physiological reference case from this study			In vivo data from the literature (Zarrinkoob et al., 2015; Correia de Verdier and Wikström, 2016; Zarrinkoob et al., 2016)		
	Vessel	Mean flow (mL min^-1^)	Flow fraction (%)	PI (−)	Mean flow (mL min^-1^)	Flow fraction (%)	PI (−)
Inflows	LICA	254.5 ± 3.1	38.0	1.29 ± 0.05	236 ± 41	36.2	0.96 ± 0.15
	RICA	247.6 ± 2.8	36.9	0.94 ± 0.03	236 ± 41	36.2	0.96 ± 0.15
	LVA	88.2 ± 1.2	13.2	1.18 ± 0.05	90 ± 17	13.8	1.11 ± 0.18
	RVA	79.9 ± 1.1	11.9	1.16 ± 0.05	90 ± 17	13.8	1.11 ± 0.18
Outflows	LACA	92.0 ± 1.0	14.4	0.45 ± 0.02	93 (28–195)	13.3	0.60 (0.18–1.57)
	RACA	92.0 ± 1.1	14.4	0.50 ± 0.03	113 (36–190)	16.2	0.67 (0.23–1.19)
	LMCA	163.7 ± 1.7	25.6	0.57 ± 0.02	169 (111–255)	24.2	0.71 (0.38–1.54)
	RMCA	165.8 ± 1.7	25.9	0.48 ± 0.02	174 (127–264)	24.9	0.69 (0.44–1.64)
	LPCA	65.6 ± 0.8	10.3	0.63 ± 0.03	77 (31–133)	11.0	0.58 (0.26–1.00)
	RPCA	60.0 ± 0.7	9.4	0.56 ± 0.03	72 (22–115)	10.3	0.56 (0.16–0.86)

### 3.1 Occlusion of the M1 segment of the left middle cerebral artery

The left M1 occlusion case, depicted in Figure 3A, resulted in a tCBF of 635.5 ± 7.0 mL min^-1^ at the MAP of 103 ± 2 mmHg with the pulse pressure of 78 ± 2 mmHg. The LPComA flow was antegrade for the ipsilateral M1 occlusion case, flowing unidirectional from LICA to LPCA (see Figures 3B–G) with 19.2 ± 2.2 mL min^-1^ and a PI value of 1.02 ± 0.18. The tCBF decreased by 2% for the M1 occlusion compared to the physiological reference case, while afferent flow fractions mainly shifted from the LICA to the RICA, decreased by 16% and increased by 14%, respectively. Efferent flow fractions in general increased by 30%–40%, while RPCA P2 increased by 15%. The measured systolic velocity peak for the M1 occlusion case was 0.51 m s^-1^, and the systolic velocity averaged over the vessel diameter was 0.28 m s^-1^.

### 3.2 Left carotid-T occlusion

The left carotid-T occlusion case, depicted in Figure 4A, resulted in a tCBF of 522.7 ± 6.4 mL min^-1^ at the MAP of 102 ± 2 mmHg with the pulse pressure of 78 ± 2 mmHg. The LPComA flow was antegrade at this condition, flowing unidirectional from LICA to LPCA (see Figures 4B–G) with 57.1 ± 5.5 mL min^-1^ and a PI value of 0.79 ± 0.14. At this condition, tCBF was decreased by 19% in comparison to the reference case. Compared to the reference case, the LICA flow fraction decreased by 70%, while the RICA flow fraction increased by 55%, and the RMCA M1 flow fraction, by 66%. The LPCA flow fraction increased by 83%, while the anterior circulation decreased by 20% and 6% for the left and right sides, respectively. The measured systolic velocity peak for the carotid-T occlusion case was 1.09 m s^-1^, and the systolic velocity averaged over the vessel diameter was 0.57 m s^-1^.

### 3.3 Basilar artery occlusion

The BA occlusion case, depicted in Figure 5A, resulted in a tCBF of 603.4 ± 6.3 mL min^-1^ at the MAP of 100 ± 2 mmHg with the pulse pressure of 77 ± 1 mmHg. The LPComA flow was antegrade at these conditions, flowing unidirectional from LICA to LPCA (see Figures 5B–G) with 24.6 ± 3.3 mL min^-1^ and a PI value of 1.68 ± 0.25. For the BA occlusion case, tCBF was decreased by 7% in comparison to the reference case, while bilateral afferent flow fractions increased uniformly. The flow fractions in the posterior circulation dropped, mainly at the LPCA, in favor of evenly increasing flow fractions at LACA and LMCA by 4% and 6%, respectively.

The mean flow and PI data on the investigated cases are summarized in Table 2, while the complete time-dependent data are available to the research community in the Supplementary Material.

**TABLE 2:** Mean flows and PI values for the total cerebral blood flow and the single arteries acquired for the three investigated cases. LPComA flow is positive for retrograde flow from LPCA to LICA and negative for the antegrade flow. The standard deviation (s.d.) indicates the precision of the 150 underlying data samples for the ultrasound flow measurements, while the s.d. of the PIV-derived LPComA flow indicates the measurement uncertainty.

	Physiological reference		LMCA M1 occlusion		Left carotid-T occlusion		BA occlusion	
	Mean flow (mL min^-1^)	PI (−)	Mean flow (mL min^-1^)	PI (−)	Mean flow (mL min^-1^)	PI (−)	Mean flow (mL min^-1^)	PI (−)
tCBF	647.4 ± 6.7	0.69 ± 0.02	635.5 ± 7.0	0.67 ± 0.02	522.7 ± 6.4	0.75 ± 0.03	603.4 ± 6.3	0.75 ± 0.02
LICA	254.5 ± 3.1	1.29 ± 0.05	208.1 ± 3.0	1.61 ± 0.08	*See limitations*	-	311.6 ± 3.9	1.21 ± 0.04
RICA	247.6 ± 2.8	0.94 ± 0.03	274.9 ± 3.3	0.85 ± 0.03	288.6 ± 3.7	0.84 ± 0.03	306.4 ± 3.5	0.94 ± 0.03
LVA	88.2 ± 1.2	1.18 ± 0.05	90.2 ± 1.3	1.14 ± 0.05	83.5 ± 1.3	1.20 ± 0.05	-	-
RVA	79.9 ± 1.1	1.16 ± 0.05	81.8 ± 1.2	1.13 ± 0.05	75.5 ± 1.1	1.18 ± 0.05	-	-
LACA	92.0 ± 1.0	0.45 ± 0.02	116.1 ± 1.4	0.43 ± 0.02	58.5 ± 0.7	0.56 ± 0.04	88.9 ± 1.0	0.48 ± 0.02
RACA	92.0 ± 1.1	0.50 ± 0.03	124.0 ± 1.6	0.53 ± 0.02	68.8 ± 1.1	0.50 ± 0.04	86.6 ± 1.1	0.46 ± 0.03
LMCA	163.7 ± 1.7	0.57 ± 0.02	-	-	-	-	160.5 ± 1.8	0.60 ± 0.02
RMCA	165.8 ± 1.7	0.48 ± 0.02	225.9 ± 2.6	0.48 ± 0.01	218.2 ± 2.6	0.50 ± 0.02	152.8 ± 1.6	0.44 ± 0.02
LPCA	65.6 ± 0.8	0.63 ± 0.03	89.3 ± 1.1	0.61 ± 0.02	95.1 ± 1.3	0.61 ± 0.02	52.5 ± 0.7	0.58 ± 0.03
RPCA	60.0 ± 0.7	0.56 ± 0.03	66.9 ± 0.9	0.55 ± 0.03	66.0 ± 0.9	0.57 ± 0.02	52.4 ± 0.7	0.47 ± 0.03
LPComA	12.0 ± 0.9	0.63 ± 0.10	−19.2 ± 2.2	1.02 ± 0.18	−57.1 ± 5.5	0.79 ± 0.14	−24.6 ± 3.3	1.68 ± 0.25

## 4 Discussion

The objective of this study was to demonstrate the feasibility of gaining quantitative information on the hemodynamics in the CoW, including collateral flow and pulsatility, during large vessel occlusions under controlled conditions. Pressures and flows were quantified in the afferent and efferent arteries of the CoW, and particularly, the volumetric flow and pulsatility of the PComA were determined. Transient *in vivo* data on the pathological collateral flow during large vessel occlusions are not available; still, some studies provide clinical observations that we used to examine the reliability of our cerebrovascular flow model.

The presented advanced experimental setup proved high stability and repeatability of the hemodynamic conditions due to very low tCBF standard deviations of <4% over 150 cardiac cycles, ensuring precise LPComA flow measurements within the cerebrovascular phantom.

### 4.1 Reliability of the physiological reference case

*In vitro* measurement reliability depends on the capacity to reproduce a physiological setting in a stable and reproducible manner. The physiological case was created by calibrating MAP, pulse pressure, tCBF, and both flow fractions and PI values of the afferent and efferent vessels of a population-averaged cerebrovascular phantom model to available *in vivo* data (Zarrinkoob et al., 2015; Correia de Verdier and Wikström, 2016; Zarrinkoob et al., 2016). The comparison of the flow fractions shown in Table 1 indicates that the flow distribution within the experimental flow model is consistent with the reported *in vivo* values. The physiological blood flow in the cerebral arteries is pulsatile and described by the PI values (Correia de Verdier and Wikström, 2016; Zarrinkoob et al., 2016), which are met by the experimental values, except for the higher LICA flow PI value. The velocity field acquired by PIV had an equidistant vector spacing of 0.07 mm through a drastically increased spatial resolution compared to a previous *in vitro* study (Luisi et al., 2023), allowing for reliable flow quantification inside the LPComA with at least 20 velocity vectors along the vessel diameter. We obtained stable and repeatable conditions proven by low standard deviations for MAP, pulse pressure, mean flows, and PI (see Table 2). Under these controlled conditions, LPComA resulted to be retrograde with a peak flow velocity of 0.18 m s^-1^. This finding is in accordance with time-resolved three-dimensional phase contrast magnetic resonance imaging of a complete CoW anatomy (van Ooij et al., 2013) and color-coded duplex sonography measurements in patients with bilateral PComA, which showed a retrograde flow direction in the vast majority of normal control subjects at higher flow velocities without further specification of CoW anatomy completeness (Klotzsch et al., 1996). In summary, clinically reported values could be confirmed in the presented *in vitro* setup, and the resolution of the acquisition setup with a simultaneous hemodynamic data recording supports the reliability of the presented LPComA flow measurement.

### 4.2 Interpretation of M1 and carotid-T occlusion cases

After both M1 and carotid-T occlusions, the initially retrograde LPComA flow direction was inverted. The observed flow rerouting in the ipsilateral PComA during the MCA M1 occlusion is in accordance with findings from *in vivo* measurements obtained through angiography (Liebeskind et al., 2016). Although MAP and pulse pressure remained stable after inducing the occlusions, the higher systemic resistance decreased the tCBF by 2% and 19% from M1 to carotid-T occlusion, respectively. The afferent flow was shifted to the contralateral side of the occlusion in both cases, which, in turn, increased the contralateral MCA flow by approximately 30% compared to the reference. Flow shifting toward the contralateral side of the occluded M1 segment is in line with findings of an *in vitro* study on a complete CoW phantom (Fahy et al., 2015). However, tCBF is reduced by the occlusion, confirming previous observations in a setup with the aortic bypass flow (Luisi et al., 2023). In case of the left M1 occlusion, a path to the anterior circulation leads through the LACA A1 segment, rerouting the carotid flow to the anterior circulation and increasing it by approximately 30%. In case of the carotid-T occlusion, the AComA vascular resistance limits the flow that can be rerouted from the contralateral side into the ipsilateral ACA. A mean contralateral RPComA flow at this condition is estimated to be 57 mL min^-1^, flowing from the posterior to the anterior circulation. In addition to the inversion of LPComA flow direction, the volumetric flow rate is increased with the severity of the occlusion.

### 4.3 Interpretation of the BA occlusion case

The collateral flow of LPComA was inverted after the BA occlusion to an antegrade flow. Under these conditions, tCBF is reduced by 7% compared to the physiological reference case; however, flow fractions in the posterior circulation were reduced by only 6%–14% despite the complete BA occlusion. *In vivo* studies with a cohort of 102 patients with BA occlusion showed that the posterior circulation Alberta stroke program early CT score (PC-ASPECTS) was on average 9 of 10 (Heit et al., 2023), indicating, for the most part, an absence of visible posterior circulation ischemia (Puetz et al., 2008). Our experimental results confirm the *in vivo* results from an exclusively hemodynamic perspective within the CoW. The support of the posterior circulation is due to an increased collateral flow in our case, which additionally causes the RPCA P1 flow to be inverted. We explain this by the fact that the RPComA diameter was larger and the vessel was shorter with less curvature compared to the LPComA in the cerebrovascular phantom model analyzed here, which results in a lower vascular resistance in the RPComA compared to the left side. The consequently larger RPComA flow originating from the RICA is thus shunted through the RPCA P1 segment to the left side of the posterior circulation.

### 4.4 Limitations

The objective of this study was achieved using a single cerebrovascular anatomy at a specific physiological state; thus, generalization of the *in vitro* results to arbitrary *in vivo* cases is limited. It is necessary to note that the comparison between the occlusion cases in this study and the *in vivo* findings from the literature is qualitative, and therefore, the *in vitro* data provided should be handled with caution. However, this advanced experimental setup allows the investigation of any CoW anatomy at various clinical conditions. The investigated cerebrovascular phantom excluded smaller arteries such as the ophthalmic artery, which makes up for approximately 2% of the tCBF (Zarrinkoob et al., 2015). For the occlusion cases, in which the antegrade LPComA flow was observed, the absence of the ophthalmic artery flow in the presented cerebrovascular phantom could potentially overestimate the LPComA flow. Although the PIV measurement technique did not allow measuring all collateral arteries simultaneously due to optical restrictions, we have shown the feasibility of the transient flow measurement within the LPComA, the smallest vessel segment in our cerebrovascular phantom. In the carotid-T occlusion case, the LICA flow dropped below the linearity range of the flow sensor; thus, the flow value had an unspecified accuracy, for which reason we excluded the LICA mean flow and PI values from Table 2. However, mean LICA and LPComA flows have to be the same; thus, the LPComA flow value can replace the mean LICA flow for the carotid-T occlusion case. The complex mechanisms of autoregulation associated with vessel constriction and dilation, as well as the role of the distal leptomeningeal collaterals, are not replicated in this cerebrovascular flow model. For example, in the left carotid-T occlusion case, the anterior circulation is undersupplied. In reality, this deficiency could be potentially counteracted by vessel dilation, while increased RMCA and LPCA flows could lead to vessel constriction, depending on the local pressure change within the vessels triggering the mechanism. This flow resistance adaptation in the cerebral arteries and arterioles affects the overall hemodynamics in a way that cannot be investigated with the current setup. The uncertainty of the fluid properties within the temperature range was less than 3%, and consequently, the potential influence is considered low for this study.

### 4.5 Conclusion

This study introduced an advanced experimental setup for gaining detailed insights into time-dependent cerebral hemodynamics within anatomical cerebrovascular phantom models during large vessel occlusions that are currently not available *in vivo*. We investigated a physiological baseline case, as well as MCA M1, carotid-T, and BA occlusions, and provided quantitative hemodynamic measurements of collateral flows within a cerebrovascular phantom. The experimental results were compared with clinical findings, demonstrating the capability of this realistic cerebrovascular flow model. Detailed information on this study is provided to the research community for the validation of *in silico* models. In order to optimize blood flow control during the thrombectomy procedure, factors such as the heart rate and systemic blood pressure have to be considered, which can be modeled with this experimental setup. For analyzing patient-specific anatomies, respective phantom models need to be manufactured prior to investigation in this setup. In summary, the introduced realistic cerebrovascular flow model opens the possibility for investigating different topics in neurovascular interventions in controlled preclinical laboratory studies under various clinical conditions.

## Data Availability

The original contributions presented in the study are included in the article/Supplementary Material; further inquiries can be directed to the corresponding author.
